# Antibiotic resistance and typing of the methicillin-resistant *Staphylococcus aureus* clones in Kuwait hospitals, 2016–2017

**DOI:** 10.1186/s12866-020-02009-w

**Published:** 2020-10-16

**Authors:** Samar S. Boswihi, Edet E. Udo, Wadha AlFouzan

**Affiliations:** Department of Microbiology, Faculty of Medicine, Kuwait University, Kuwait City, Kuwait

**Keywords:** MRSA, DNA microarray, Antibiotic resistance, Genotypes, Clonal complex

## Abstract

**Background:**

Methicillin-resistant *Staphylococcus aureus* (MRSA) belong to diverse genetic backgrounds that differ in antibiotic resistance. Knowledge of the local clonal composition of MRSA strains is important for patients’ management and for designing effective control and eradication methods. The aim of this study was to compare the antibiotic resistance patterns and genotypic characteristics of MRSA isolates obtained in public hospitals in Kuwait in 2016 and 2017 for changes in their resistance patterns and clonal composition.

**Methods:**

A total of 4726 MRSA isolates obtained in 2016–2017 from clinical specimens in Kuwait public hospitals were characterized using antibiogram, SCC*mec* typing, *spa* typing and DNA microarray.

**Results:**

The isolates expressed resistance to fusidic acid (52.9%), kanamycin (41.6%), gentamicin (32.5%) and erythromycin (36.2%). The prevalence of high-level mupirocin resistance decreased from 3.7% in 2016 to 2.4% in 2017, while the proportion of resistance to other antibiotics remained relatively stable. A total of 382 *spa* types were detected with eight *spa* types, t688 (*N* = 547), t304 (*N* = 428), t860 (*N* = 394), t127 (*N* = 306), t044 (*N* = 230), t311 (*N* = 243), t223 (*N* = 184) and t002 (*N* = 181) constituting 53.1% of the MRSA isolates in 2016–2017. Of the 3004 MRSA isolates obtained in 2016 (*N* = 1327) and 2017 (*N* = 1677) selected for DNA microarray analysis, 26 clonal complexes (CCs) were identified. Most of the isolates belonged to CC1 (*N* = 248), CC5 (*N* = 833), CC6 (*N* = 241), CC8 (*N* = 292), CC22 (*N* = 421), CC30 (*N* = 177), CC80 (*N* = 177) and CC97 (*N* = 171). The prevalence of CC5 isolates has significantly (*p* ≤ 0.05) increased from 294 isolates in 2016 to 539 isolates in 2017. Although CC22 increased from 196 isolates in 2016 to 225 isolates in 2017, CC1 increased from 112 isolates in 2016 to 136 isolates in 2017, CC6 increased from 103 isolates in 2016 to 138 isolates in 2017, these changes were not significant (*p* ≥ 0.05).

**Conclusion:**

These results revealed the diversity in the genetic backgrounds of MRSA isolates and the stable maintenance of the dominant MRSA clones in Kuwait hospitals in 2016 and 2017 suggesting an on-going transmission of these clones. Novel and creative infection prevention and control measures are required to curtail further transmission.

**Supplementary information:**

**Supplementary information** accompanies this paper at 10.1186/s12866-020-02009-w.

## Background

Since its report in 1961 in England [1], methicillin-resistant *Staphylococcus aureus* (MRSA) has spread to many countries causing serious infections that are sometimes difficult to treat [2]. Initially, MRSA was a well-established pathogen among elderly patients with previous admission to healthcare settings and with history of previous antibiotic usage. This type of MRSA was known as healthcare-associated or healthcare-acquired MRSA (HA-MRSA) [3]. Then in the 1990’s, a new lineage of MRSA emerged in people with no previous history of hospitalization or exposure to the healthcare system and previous antibiotic use which was designated as community-associated or community-acquired MRSA (CA-MRSA) [4].

Molecular epidemiological typing techniques, such as SCC*mec* typing, staphylococcal protein A (*spa*) typing, multilocus sequence typing (MLST), pulsed-filed gel electrophoresis (PFGE) and DNA microarray have been used to study the genetic background of MRSA. HA-MRSA isolates carry relatively large SCC*mec* genetic element belonging to type I, II, or III, and are usually resistant to multiple non-beta-lactam antibiotics [2]. In contrast, CA-MRSA isolates carry smaller sized SCC*mec* elements belonging to SCC*mec* type IV, V or VI and are usually sensitive to most non-beta-lactam antibiotics [2]. MRSA isolates carrying different SCC*mec* genetic elements have been further differentiated using MLST and eBURST which grouped them into sequence types and clonal complexes (CCs) [2].

Studies have shown that CA-MRSA have replaced HA-MRSA in many countries including Kuwait [5], Singapore [6], United Arab Emirates [7] and Portugal [8]. A previous study in Kuwait showed that the clonal composition of the MRSA has changed significantly from 1992 to 2010 [5] with the emergence of different CA-MRSA clones. The same report revealed that CC8/ST239-III remained the most common clone in Kuwait hospitals from 1992 to 2010, although their prevalence decreased overtime [5]. During the same period, the prevalence of CA-MRSA clones including CC5/ST5-IV/V, CC80/ST80-IV and ST1-IV/V were increasing [5]. To provide an update on the clonal composition of MRSA strains circulating in Kuwait hospitals, this study compared the antibiotic resistance patterns and genotypic characteristics of MRSA isolates obtained in public hospitals in Kuwait in 2016 and 2017 for changes in clonal composition and resistance profile.

## Results

### Specimen source and antibiotic susceptibility profile of MRSA isolates

MRSA isolates obtained in 2016 and 2017 were mostly from skin and soft tissue infection specimens (1983; 41.9%). This was followed by nasal swabs (1276; 26.9%), endotracheal specimens (271; 5.7%), blood (200; 4.2%), urine (102; 2.1%), throat (89; 1.8%) and ear (64; 1.3%). The clinical sources for 741 (15.6%) of the isolates were not provided.

The distribution of resistance phenotypes for all MRSA isolates obtained in 2016 and 2017 is shown in Table 1. All isolates were sensitive to vancomycin (MIC: ≤ 2 μg/ml), teicoplanin (MIC: ≤ 2 μg/ml) and linezolid. Besides beta-lactam resistance, most of the MRSA isolates obtained in 2016 and 2017 were resistant to fusidic acid (52.9%). This was followed by resistance to kanamycin (41.6%), tetracycline (37.5%), erythromycin (36.2%), trimethoprim (35.8%), ciprofloxacin (35.7%), gentamicin (32.5%), clindamycin (16.9%), and chloramphenicol (14.1%). Only a small proportion of the isolates expressed high-level resistance (HLR) to mupirocin (3.1%).

**Table 1:** Distribution of resistance phenotype among MRSA isolates in 2016–2017

Resistance phenotype	2016 (*N* = 2305)		2017 (*N* = 2421)		Total (*N* = 4726)		*p*-value
	No.	%	No.	%	No.	%	
PG	2118	92	2363	98	4481	94.8	
GM	763	33	775	32	1538	32.5	
KM	974	42	992	41	1966	41.6	
EM	912	40	800	33	1712	36.2	**≤ 0.05**
CC(I)	435	19	366	15	801	16.9	**≤ 0.05**
CC(C)	404	18	365	15	769	16.2	**≤ 0.02**
CM	290	13	376	16	666	14.1	**≤ 0.05**
TET	832	36	943	39	1775	37.5	
TP	861	37	835	35	1696	35.8	
RF	12	0.5	10	0.4	22	0.46	
FA	1178	51	1324	55	2502	52.9	
CIP	854	37	835	35	1689	35.7	
MUP(L)	189	8	221	9	410	8.6	
MUP(H)	**87**	**3.7**	**59**	**2.4**	**146**	**3.1**	**≤ 0.05**

*Abbreviations*: *PG* penicillin G, *Gm* gentamicin, *Km* kanamycin, *Tet* tetracycline, *Em* erythromycin, *CC (I)* induced-resistance clindamycin, *CC (C)* constitutive-resistance clindamycin, *Tp* trimethoprim, *RF* rifampicin, *Fa* fusidic acid, *CM* chloramphenicol, *Cip* ciprofloxacin, *MUP (H)* mupirocin high- level resistance, *MUP (L)* mupirocin low- level resistance

Comparison of the distribution of antibiotic resistance in MRSA isolates obtained between 2016 and 2017 is shown in Table 1. During 2016–2017, there were significant (*p* ≤ 0.05) decrease in the prevalence of resistance to erythromycin, clindamycin and high-level mupirocin resistance, while resistance to chloramphenicol increased significantly (Table 1).

Results of DNA microarray analysis revealed that the high-level mupirocin-resistant isolates were positive for *mupA* that encodes an alternative isoleucyl-tRNA synthetase (*ileS2)* which is unaffected by mupirocin. Similarly, resistance to gentamicin, erythromycin and clindamycin, and tetracycline corresponded with the presence of their respective determinants, *aacA-aphD*, *erm(A)/erm(C),* and *tet(K)*/*tet(M).* Fusidic acid resistance was mediated by *fusC* in most of the isolates and by *fusB/faR1* in CC80 isolates.

### Prevalence of SCC*mec* types in MRSA isolates in 2016–2017

The dominant SCC*mec* type among the MRSA isolates obtained in 2016–2017 was SCC*mec* type IV (47.0%) (Table 2). This was followed by SCC*mec* type V (29.8%), type III (12.1%) and type VI (9.3%). SCC*mec* type II (0.5%) and type I (0.08%) were detected in small numbers. Thirty-four isolates carried a new combination of SCC*mec* types (SCC*mec* IV + V).

**Table 2:** Distribution of SCC*mec* types among MRSA isolates in 2016–2017

SCC*mec* types	2016		2017		Total		*p*-value
	No.	%	No.	%	No.	%	
I	1	0.04	3	0.1	4	0.08	
II	14	0.6	10	0.4	24	0.5	
III	291	13	282	11.7	573	12.1	
IV	1148	50	1077	44.5	2225	47.0	**≤ 0.05**
V	701	30	708	29	1409	29.8	
VI	170	7	273	11.2	443	9.3	**≤ 0.05**
IV + V	12	0.5	22	1	34	0.7	
ND	–	–	46	2	46	0.9	

There were no significant changes in the distribution of SCC*mec* types I, II, III and V in MRSA isolates between 2016 and 2017 (Table 2). MRSA isolates carrying type VI increased (*p* ≤ 0.05) from 7% in 2016 to 11.2% in 2017, while those carrying SCC*mec* type IV decreased (*p* ≤ 0.05) from 50% in 2016 to 44.5% in 2017 (Table 2).

### Prevalence of *spa* types among MRSA isolates in 2016–2017

In total, 382 *spa* types were identified among the MRSA isolates obtained in 2016–2017. The distribution of the common *spa* types among the isolates obtained in 2016 and 2017 is shown in Table 3. *Spa* type t688 (23%) was the dominant *spa* type detected in both years. This was followed by t304, t860, t127, t044, t311, t002 and t223 (Table 3). In addition, 354 *spa* types were detected in less than 10 isolates. *Spa* types could not be assigned for 118 isolates in both years.

**Table 3:** Distribution of *spa* types among MRSA isolates in 2016–207

*Spa* types	2016		2017		Total		*p*-value
	No.	%	No.	%	No.	%	
t688	240	10.4	307	12.6	547	23	**≤ 0.05**
t304	211	9.1	217	8.9	428	18	
t860	190	8.2	204	8.4	394	16.6	
t127	151	6.5	155	6.4	306	12.9	
t044	132	5.7	98	4	230	9.7	**≤ 0.05**
t311	124	5.3	119	4.9	243	10.2	
t002	103	4.4	78	3.2	181	7.6	**≤ 0.05**
t223	94	4	90	3.7	184	7.7	
t267	65	2.8	68	2.8	133	5.6	
t019	58	2.5	58	2.3	116	4.8	
t3841	54	2.3	44	1.8	98	4.1	
t084	38	1.6	55	2.2	93	3.8	
t852	35	1.5	24	0.99	59	2.49	
t945	33	1.4	21	0.86	54	2.26	
t105	27	1.17	20	0.82	47	1.99	
t032	26	1.12	19	0.78	45	1.9	
t657	24	1	23	0.95	47	1.95	
t359	18	0.78	32	1.3	50	2.08	
t786	17	0.73	8	0.33	25	1.06	
t16187	15	0.65	11	0.45	26	1.1	
t008	14	0.6	21	0.86	35	1.46	
t037	14	0.6	11	0.45	25	1.05	
t018	12	0.52	4	0.16	16	0.68	
t021	12	0.52	14	0.57	26	1.09	
t701	12	0.52	7	0.28	19	0.8	
t11822	11	0.47	5	0.2	16	0.67	
t421	11	0.47	17	0.7	28	1.17	
t315	10	0.43	12	0.49	22	0.92	
t535	10	0.43	11	0.45	21	0.88	
ND	7	0.3	111	4.5	118	4.8	

*Spa* types detected in < 10 isolates: t024, t463, t1200, t2121, t5708, t026, t7011, t10659, t13697, t17282, t985, t4407, t2849, t045, t5634, t1339, t2518, t680, t068, t7139, t10795, t14230, t17649, t5562, t454, t310, t12219, t790, t16360, t2720, t7200, t086, t729, t10888, t1427, t1816, t5608, t4557, t3175, t1247, t9228, t16861, t279, t7348, t088, t7342, t1120, t14392, t1830, t5673, t4565, t3379, t1309, t9867, t17330, t3107, t8731, t091, t7358, t11288, t14838, t186, t570, t4867, t3562, t132, t012, t1752, t3235, t878, t094, t7466, t114, t15181, t2164, t5704, t4892, t3782, t2526, t062, t17556, t334, t902, t10028, t747, t11714, t15435, t217, t578, t4955, t3825, t4019, t10002, t177, t345, t934, t10094, t774, t118, t16302, t2177, t582, t4981, t3967, t442, t10892, t189, t4018, t954, t10234, t777, t11836, t16470, t2235, t605, t5045, t398, t4549, t975, t203, t425, t9606, t10306, t8369, t121, t16606, t2413, t6071, t5146, t416, t1062, t9042, t1317, t17117, t258, t1034, t853, t122, t16877, t242, t622, t525, t422, t17281, t9207, t681, t17275, t2658, t10347, t8657, t12413, t16945, t2467, t6584, t537, t4336, t2790, t9448, t6845, t17279, t2672, t10395, t8962, t12743, t16946, t2529, t6675, t5414, t437, t10422, t903, t13158, t17084, t2571, t6769, t547, t4403, t046, t1154, t1198, t1215, t138, t16185, t1839, t2601, t3364, t362, t4326, t8154, t010, t014, t1028, t11863, t1252, t131, t14228, t14700, t1548, t16182, t16186, t16202, t1977, t211, t2393, t3012, t3243, t3387, t355, t3896, t4045, t4724, t521, t5485, t579, t639, t693, t8221, t845, t8506, t9673, t004, t016, t034, t050, t067, t10116, t10118, t1039, t10405, t1081, t10836, t11113, t11206, t11462, t1147, t1175, t11901, t12068, t12236, t12398, t12537, t13429, t13699, t1379, t14090, t144, t1476, t1504, t15069, t15224, t1556, t15778, t15801, t1588, t1593, t16181, t1635, t16361, t16373, t16468, t16486, t16549, t16578, t16579, t16604, t16605, t16901, t16903, t16904, t16905, t1836, t1855, t190, t1965, t1991, t214, t2251, t228, t253, t2622, t2734, t2770, t2802, t2933, t3010, t3092, t3196, t321, t324, t325, t330, t3494, t3651, t4223, t4224, t4359, t450, t504, t527, t541, t5593, t5772, t586, t591, t5994, t6258, t6670, t6693, t6827, t711, t713, t743, t7604, t7623, t7656, t7685, t779, t8009, t8166, t8168, t8348, t8934, t899, t9231, t9434, t948, t9638

A comparison of the distribution of *spa* types of MRSA isolates obtained in 2016–2017 presented in Table 3 revealed that the prevalence of *spa* type t688 increased (*p* ≤ 0.05) from 10.4% in 2016 to 12.6% in 2017, while the proportions of t002 and t044 were significantly decreased during the 2 years. No significant changes were observed among the other major *spa* types. Some sporadic *spa* types were observed in isolates obtained either in 2016 or 2017 as shown in the supplementary Table S1. The association of *spa* types with specific genotypes is presented in Table S1.

### Distribution of MRSA clones in 2016–2017

The clonal complexes (CCs) of 3004 MRSA isolates obtained in 2016 (*N* = 1327) and 2017 (*N* = 1677), selected on the basis of *spa* types was determined using DNA microarray. The selection included all clinical samples from different hospitals with the same *spa* type.

Twenty-six clonal complexes (CCs) were obtained in both years. The clonal complexes (CCs) were CC1, CC5, CC6, CC7, CC8, CC9/ST834, CC15, CC22, CC30, CC45, CC49, CC80, CC88, CC96, CC97, CC121, CC152, CC361, CC398, CC509, CC779, CC913, CC1153, CC2198, CC2250/2277 and CC2596. In addition, three sequence types, ST59, ST72 and ST2867 were identified. The distribution of the MRSA clones is shown in supplementary Table (Table S1). The dominant clonal complexes identified in both years were CC5 (833 isolates), CC22 (421 isolates), CC8 (292 isolates), CC1 (248 isolates), CC6 (241 isolates), CC30 (177 isolates), CC80 (177 isolates) and CC97 (171 isolates) (Table S1). The other clonal complexes including CC7, CC9/ST834, CC15, CC45, CC49, CC88, CC96, CC121, CC152, CC361, CC398, CC509, CC779, CC913, CC1153, CC2198, CC2250/2277, CC2596 and the three sequence types ST59, ST72 and ST2867 were less frequently detected among MRSA isolates during 2016 and 2017 (Table S1). The composition of the major clonal complexes is presented below.

### Clonal Complex 1 (CC1)

CC1 consisted of 16 MRSA genotypes. The most common CC1 genotype was CC1-MRSA-V + SCCfus [PVL^+^] (78 isolates) which occurred in 35 isolates in 2016 and in 43 isolates in 2017. Of the 16 *spa* types identified among the CC1-MRSA-V + SCCfus [PVL^+^] isolates, the t127 was the most common *spa* type in 2016 and 2017. *Spa* types t3896, t6693, t948, t1252, t693, t591 were only detected in 2016, while t114, t1589, t16861, t177, t2207, t398, t605, t17556, t2658 were only detected in 2017. Other common genotypes of CC1 were CC1-MRSA-V + SCCfus (50 isolates) and CC1-MRSA-IV, WA MRSA-1/57 (34 isolates). Nineteen CC1-MRSA-V + SCCfus isolates were detected in 2016. This increased to 31 isolates in 2017. They consisted of eight *spa* types with *spa* types t1252 detected only in 2016 and t2720, t948, t177, t217, t2720 detected only in 2017 respectively while, *spa* types t127 and t693 were found in both years. Similarly, strains of CC1-MRSA-IV, WA MRSA-1/57 increased from nine isolates in 2016 to 25 isolates in 2017 and were associated with nine *spa* types with three *spa* types t3494, t086 and t688 detected only in 2016 and t1589, t17649, t5388 detected only in 2017. Four novel variants of CC1-MRSA were identified. These were CC1-MRSA-[V/V_T_ + fus] (PVL^+^), CC1-MRSA-PseudoSCC*mec* [classB+fus + ccrAB1], CC1-MRSA-[V/V_T_ + fus] and CC1-MRSA-[V/V_T_ + fus + ccrAB1] (PVL^+^). While eight isolates of CC1-MRSA-[V/V_T_ + fus] (PVL^+^) were detected in 2016, only four of the isolates were detected in 2017. The other genotypes, CC1-MRSA-PseudoSCC*mec*[classB+fus + ccrAB1], CC1-MRSA-[V/V_T_ + fus], and CC1-MRSA-[V/V_T_ + fus + ccrAB1] (PVL^+^) were detected only in 2016. The remaining CC1 MRSA strains are presented in Table S1.

A total of 49 isolates of ST772-MRSA-V [PVL^+^], Bengal Bay Clone were identified in 2016 (27 isolates) and 2017 (22 isolates). They consisted of seven *spa* types. *Spa* types t10795, t345, t5414, t657 were detected in 2016 and 2017 while two *spa* types, t1839 and t3387 were detected only in 2016 while t17441 was detected only in 2017. Other ST772-MRSA genotypes identified only in 2017 consisted of ST772-MRSA-V, ST772-MRSA-V [PVL^+^], Bengal Bay Clone/WA MRSA-60 [ccr mutation/deletion] and ST772-MRSA-[mec V + fus] (PVL^+^). The ST772-MRSA- [mec V + fus] (PVL^+^) strain was identified as a new variant of ST772 in 2017 and belonged to *spa* type t20638.

### Clonal Complex 5 (CC5)

CC5 consisted of 19 MRSA genotypes with CC5-MRSA-VI + SCCfus (337 isolates) as the dominant genotype. The CC5-MRSA-VI + SCCfus genotype was detected in 87 isolates in 2016 and 250 isolates in 2017 which represented a significant increase. It was associated with 11 *spa* types with t2235, t535, t688, t954 found in both years. (Table S1). The other common genotype was CC5-MRSA-V + SCCfus, WA MRSA-14/109 which slightly increased in prevalence from 79 in 2016 to 89 in 2017. CC5-MRSA-IV, [PVL^+^] and CC5-MRSA-V [sed/j/r^+^], WA MRSA-11/34/35/90/108 strains increased from 40 and 26 isolates in 2016 to 55 and 42 isolates in 2017 respectively. Four CC5 strains detected only in 2017 were CC5-MRSA-V [PVL^+^], CC5-MRSA-II [ACME^+^], WA MRSA-125 and the new variant strains CC5-MRSA-[V/V_T_ + fus] and CC5-MRSA-[II + ccrAB4]. In addition, a new CC5 variant, CC5-MRSA-[V/V_T_ + fus] (PVL^+^) was detected in 2016.

### Clonal Complex 6 (CC6)

CC6 consisted of three MRSA genotypes. One strain was identified as MSSA by DNA microarray although phenotypically resistant to cefoxitin which could be due to failure in the hybridization of *mecA* gene to the probe. The CC6-MRSA-IV, WA MRSA-51 genotype increased slightly from 100 isolates in 2016 to 136 isolates in 2017. They were associated with 27 *spa* types detected either in 2016 or 2017 (Table S1). *Spa* types t10888, t11288, t2849, t304, t701, t6845, t4403, t711, t190 were observed in CC6-MRSA-IV,WA MRSA-51 strains detected both in 2016 and 2017. Two isolates belonging to CC6-MRSA-V were detected only in 2017, while a new variant, CC6-MRSA-[IV + fus + ccrC], was only detected in 2016.

### Clonal Complex 8 (CC8)

Sixteen MRSA genotypes consisting of ST8 (*N* = 74) and ST239 (*N* = 218) were identified as CC8. The distribution of all CC8 strains is presented in Table S1. The most common CC8 genotypes were CC8-MRSA-IV [tst1^+^] (15 isolates), CC8-MRSA-IV, UK-EMRSA-14/WA MRSA-5 (13 isolates), and ST8-MRSA-IV [PVL^+^/ACME^+^], USA300 (11 isolates). The CC8-MRSA-IV [tst1^+^] genotype was detected in seven isolates in 2016 and in eight isolates in 2017, while CC8-MRSA-IV, UK-EMRSA-14/WA MRSA-5 genotype was detected in four isolates in 2016 and nine isolates in 2017. The USA300 genotype was found in two isolates in 2016 and nine isolates in 2017. The CC8 strains found only in 2016 include CC8-MRSA-IV [sea-N315^+^], CC8-MRSA-IV, [PVL^+^, sed/j/k/q/r^+^], WA MRSA-62 and CC8-MRSA-[V/V_T_ + fus] (a new variant of CC8), whereas CC8-MRSA-VI + SCCfus was identified once in 2017. Other CC8 strains were found in a small number in 2016 and 2017 (Table S1).

A total of 180 isolates were identified as ST239-MRSA-III + SCCmer, Vienna/Hungarian/Brazilian clone. This genotype was found in 101 isolates in 2016 and 79 isolates in 2017. Nine *spa* types, t1247, t1339, t15224, t6258, t713, t16187, t421, t860, t945 were associated with the ST239-MRSA-III + SCCmer, Vienna/Hungarian/Brazilian Clone in 2016, while six *spa* types, t16187, t421, t860, t945, t037, t680 were found in 2017. The distribution of the other ST239 strains is shown in Table S1.

### Clonal Complex 22 (CC22)

CC22 consisted of 12 MRSA genotypes with CC22-MRSA-IV [tst1^+^], UK-EMRSA-15/Middle Eastern variant detected in 202 isolates as the most prevalent genotype of CC22. The UK-EMRSA-15/Middle Eastern variant was detected in 89 isolates in 2016 and in 113 isolates in 2017. Another common CC22 genotype was the PVL-positive CC22-MRSA-IV (98 isolates) which was detected in 56 isolates in 2016 and in 42 isolates in 2017. The CC22-MRSA-V [fusC^+^] strain was identified once in 2017. The new variants, CC22-MRSA-IV (tst1/PVL^+^) and CC22-MRSA-[VI + fus] detected in 2016–2017 were found in 25 and four isolates respectively. Another new variant, identified as CC22-MRSA-[IV + fus + ccrAB4], appeared for the first time in 2017.

### Clonal Complex 30 (CC30)

CC30 consisted of seven ST30 and one ST36 MRSA isolates. The most common genotype was the CC30-MRSA-IV [PVL^+^], Southwest Pacific Clone (130 isolates) which was detected in 68 isolates in 2016 and in 62 isolates in 2017. The most common *spa* type identified with this genotype was t019. Three genotypes carrying SCC*mec*VI with *fusC* were identified as variants of CC30. Twelve and three isolates were identified as CC30-MRSA-[VI + fus] (PVL^+^) in 2016 and 2017 respectively. Four isolates of CC30-MRSA-[VI + fus] (PVL^+^/tst1) were detected only in 2017.

### Clonal Complex 80 (CC80)

PVL-positive and PVL-negative variants of CC80-IV-MRSA were identified in 177 isolates in 2016–2017. The CC80-MRSA-IV [PVL^+^], European CA-MRSA Clone (138 isolates) was prevalent among MRSA isolates in 2016 (62 isolates) and 2017 (76 isolates). *Spa* types t044, t042, t005, t11863, t16186, t3196, t376, t10892, t1200, t15435, t416, t1247, t131, t203, t639 were identified in the PVL-positive CC80-MRSA-IV isolates with t044 (95 isolates) as the most common *spa* type in 2016 and 2017. The PVL-negative CC80-MRSA-IV variant (36 isolates) was found in 13 isolates in 2016 and in 23 isolates in 2017. The CC80-MRSA-(truncated/atypical SCC*mec*) was identified in two PVL-positive isolates in 2016 and in one PVL-negative isolate in 2017.

### Clonal Complex 97(CC97)

A total of 171 isolates were identified as CC97 with 140 isolates recognized as CC97-MRSA-V [fusC^+^]. The other genotypes of CC97, CC97-MRSA-IV, WA MRSA-54/63 and CC97-MRSA-V were found in 11 and 15 isolates respectively in 2016–2017.

## Discussion

This study investigated antibiotic resistance and clonal composition of MRSA isolates obtained from patients in Kuwait public hospitals in 2016–2017. The results revealed some changes in the prevalence of resistance to some antibiotics over the 2 years. Significantly, the prevalence of high-level mupirocin resistance decreased from 3.7% in 2016 to 2.4% in 2017. This is consistent with previous report of low prevalence of high-level mupirocin resistance in Kuwait hospitals in recent years [9]. This is reassuring because it indicates that mupirocin can still be used successfully to control MRSA infections. On the other hand, the prevalence of fusidic acid resistance increased from 51% in 2016 to 55% in 2017 which was consistent with previous reports of the high prevalence of fusidic acid resistance in Kuwait hospitals [9]. High prevalence of fusidic acid resistance in MRSA isolated in Kuwait has been suggested to be due to several factors including the emergence or importation of many MRSA genotypes that are resistant to fusidic acid in Kuwait hospitals [10], and the extensive use of topical fusidic acid creams that are readily available over the counter without prescription in Kuwait.

Most of the MRSA isolates in this study carried SCC*mec* IV, V and VI indicating that the majority of the isolates belonged to the community-associated MRSA genotypes. The significant increase in the proportion of isolates carrying SCC*mec* VI from 2016 to 2017 could be explained by the increase in the number of CC5 isolates with SCC*mec* VI.

The majority of the isolates obtained in the 2 years belonged to *spa* types t688, t304, t860, t127, t044, t311, t223 and t002 that were also dominant previously in Kuwait hospitals [5]. Most of the CC5 isolates detected in this study were of *spa* type t688. In contrast, t002 was the dominant *spa* type of CC5-MRSA obtained in New Zealand [11], Switzerland [12] and Canada [13]. However, similar to the findings of this study, t688 is also a common *spa* type among MRSA isolates reported in Egypt [14], a country with a large population of expatriates in Kuwait.

The established eight dominant MRSA clones consisting of CC1, CC5, CC6, CC8/ST239, CC22, CC30, CC80 and CC97 [15–19] were also the dominant clones in Kuwait hospitals in 2016 and 2017 and have been present in Kuwait since the early 2000’s [5] indicating that these clones are now well established in Kuwait hospitals.

CC5 is one of the dominant and widely spread MRSA clones reported worldwide [15–19]. In this study, the CC5-MRSA isolates significantly increased (*p* ≤ 0.05) from 294 isolates in 2016 to 539 isolates in 2017 which could be due to the introduction of new CC5 strains in 2017.

We detected CC5-MRSA-II [ACME^+^] genotype for the first time in Kuwait in this study. The strain is similar to the pandemic ST5-MRSA-II clone that was reported previously in China and USA [19]. In addition, other CC5 genotypes, including CC5-MRSA-IV + SCCfus, Maltese Clone, CC5-MRSA-IV, [PVL^+^/edinA^+^], WA MRSA-121, and CC5-MRSA-V + SCCfus, WA MRSA-14/109, reported in this study were also reported in Saudi Arabia [20–22] suggesting that these clones maybe common in the Arabian Peninsula.

The study revealed a significant reduction in the proportion of CC8 and CC30 isolates in Kuwait hospitals during the study period. CC8 (ST239-MRSA-III) was the predominant clone among MRSA isolates obtained in Kuwait hospitals in the 90’s but its prevalence has since reduced [5]. The low prevalence of ST239-MRSA-III isolates observed in both 2016 and 2017 confirms its decline as a major contributor to MRSA infections in Kuwait hospitals although it is still a major cause of health-associated infections elsewhere [23].

The USA300 (ST8-MRSA-IV [PVL^+^/ACME^+^]) clone, the dominant CA-MRSA clone in North America [24] was detected for the first time in Kuwait in 2010 [5]. Although the number of USA300 isolates detected in 2017 (*N* = 9) remained remarkably low, it represents a significant increase on the single isolate obtained in 2010. As the USA300 is an important cause of infections, it is important to monitor its prevalence among patients in Kuwait hospitals.

The CC22-MRSA-IV is a well-known epidemic MRSA clone that emerged in the United Kingdom in the early 1990s [25] and soon became prevalent in other European countries [26–28]. Although CC22-MRSA was the second most common clonal complex in this study (421 isolates) similar to results of a previous study in Kuwait [5], their genotypes have increased from three genotypes identified in 2005 and 2010 [29] to 11 different genotypes in 2016 and 2017 with the CC22-MRSA-IV [tst1^+^], UK-EMRSA-15/Middle Eastern variant still the most common genotype. The tst-positive ST22-MRSA-IV, Middle Eastern variant is also common in Saudi Arabia [21], Gaza strip [30] and Jordan [31]. In contrast, there were no remarkable changes in the distribution of the other CC22 MRSA variants, CC22-MRSA-IV (tst1/PVL^+^), CC22-MRSA-[VI + fus], CC22-MRSA-[IV + fus + ccrAB4] in 2016–2017.

The proportion of isolates belonging to CC80-MRSA-IV and CC97-MRSA-V remained stable in 2016 and 2017. However, new *spa* types were seen for the first time in 2017 associated with these strains suggesting changes in their genetic composition. Furthermore, 45 isolates belonging to CC1, CC5, CC8, CC22, CC45, CC88, CC9/ST834 and ST72 carried *spa* types in 2016 that were different from those obtained in 2017 (Table S1). For example, the three isolates belonging to CC1-MRSA-V [PVL^+^] obtained in 2016 were associated with t127, t321, t386 while one isolate obtained in 2017 was associated with t2720. The CC9/ST834-MRSA-IV, WA MRSA-13 isolate obtained in 2016 was associated with t1379, while in 2017 it was associated with t1830. CC88-MRSA-V isolate obtained in 2016 was associated with t3153, while in 2017 it was associated with t6769. Also, ST72-MRSA-V, WA MRSA-91 was associated with t3092 in 2016 but with t537 in 2017. These observations suggest that the isolates obtained in both years were different although they belonged to the same clonal complex. This may impact proper management of infection caused by these isolates. Similar observation was seen in isolates belonging to CC5-MRSA-IV, Pediatric clone, CC8-MRSA-IV [sea^+^], Lyon Clone/UK-EMRSA-2, CC8-MRSA-V, WA MRSA-115/− 132, ST8-MRSA-IV [PVL+/ACME-], CC22-MRSA-[VI + fus], CC22-MRSA-IV + V [PVL^+^], CC45/agrIV-MRSA-IV, WA MRSA-23, and CC88-MRSA-[IV + fus] (Table S1).

## Conclusion

The study revealed the diversity in the genetic backgrounds of MRSA isolates and the stable maintenance of the dominant MRSA clones in Kuwait hospitals in 2016–2017 suggesting an on-going transmission of these clones. It also demonstrated the emergence of new variants of known genotypes in Kuwait hospitals in 2016 and 2017. Novel and creative infection prevention and control measures are required to curtail further transmission. It is still not clear why some MRSA clones are able to persist while others fail to survive in the healthcare environment. This warrants further investigations to identify the factors that contribute to the spread and maintenance of the successful MRSA clones.

## Methods

### Sample collection

In total, 4726 single patients, MRSA isolates were obtained from different clinical samples submitted to the clinical Microbiology diagnostic laboratory in 11 Public hospitals in Kuwait in 2016 (*N* = 2305) and 2017 (*N* = 2421). The isolates were identified using biochemical tests and tube coagulase at the diagnostic microbiology laboratory. Once it was identified as MRSA in the diagnostic laboratories, the isolates were sent to the MRSA Reference Laboratory located in the Department of Microbiology, Faculty of Medicine, Kuwait University for molecular typing where they were retested and confirmed as MRSA. The isolates were sub-cultured twice on brain-heart infusion agar (BHIA) plates to obtain pure colonies and incubated at 35 °C for 18 h. Pure cultures were preserved in beads and stored at − 20 °C and − 80 °C. They were recovered on brain-heart infusion agar (BHIA) and incubated at 35 °C prior to further testing.

### Antibiotic susceptibility testing

Susceptibility to penicillin G, gentamicin, kanamycin, erythromycin, clindamycin, tetracycline, fusidic acid, trimethoprim, mupirocin, ciprofloxacin, chloramphenicol, rifampicin, cefoxitin, linezolid, vancomycin and teicoplanin were tested using the disc diffusion method according to the Clinical Laboratory Standards Institute (CLSI) [32]. Susceptibility to cefoxitin, vancomycin, teicoplanin, and mupirocin were confirmed by minimum inhibitory concentration (MIC) determination with Etest strips (BioMerieux, Marcy l’Etoile, France) according to the manufacturer’s instructions. *S. aureus* strain ATCC25923 and ATCC29213 were used as quality control strains for the disc diffusion and MIC determination, respectively. Susceptibility to fusidic acid was interpreted according to the British Society to Antimicrobial Chemotherapy (BSAC) [33].

### Staphylococcal cassette chromosome *mec* (SCC*mec*) typing

SCC*mec* typing was performed using PCR for all MRSA isolates. Six types of SCC*mec* was determined by multiplex PCR using primers and protocols published previously [34]. Five μl of the PCR product was analyzed by 1.5% agarose gel electrophoresis to confirm amplification. Five *S. aureus* strains represented by COL (SCC*mec* I), XU642 (SCC*mec* II), WBG 525 (SCC*mec* III), WBG 9465 (SCC*mec* IV), WBG 8318 (SCC*mec* V) were used as quality control for each SCC*mec* type. The SCC*mec* types of the isolates were also derived from DNA microarray analysis.

### Staphylococcal protein a (*spa*) typing

All MRSA isolates were investigated by *spa* typing. Amplification of *spa* gene was performed using synthetic primers previously published [35]. The PCR protocol consisted of an initial denaturation at 94 °C for 4 min, followed by 25 cycles of denaturation at 94 °C for 1 min, annealing at 56 °C for 1 min, and extension for 3 min at 72 °C, and a final cycle with a single extension for 5 min at 72 °C. Five μl of the PCR product was analyzed by 1.5% agarose gel electrophoresis to confirm amplification. The amplified PCR product was purified using MicroElute Cycle-Pure Spin kit (Omega Bio-tek, Inc. USA) and the purified DNA was then used for sequencing PCR. The sequencing PCR product was then purified using Ultra-Sep Dye Terminator Removal kit (Omega Bio-tek, Inc. USA). The Purified DNA was sequenced in an automated 3130 × 1 genetic analyzer (Applied Biosystem, USA). The sequence of *spa* gene was analyzed using the Ridom Staph Type software (Ridom GmbH, Wurzburg, Germany). The software detected the *spa* repeat and assigned each isolate with *spa* type.

### DNA microarray

Based on *spa* typing, representative MRSA isolates obtained in 2016 and 2017 were subjected to DNA microarray to determine their clonal complex (CC) using the *S. aureus* Genotyping kit 2.0 (Alere, GmbH, Germany) with a protocol provided by the manufacturer [36].

### Statistical analysis

To determine if the difference in the distribution of the isolates obtained in 2016 and 2017 is statistically significant, 2-tailed Chi square and Fisher exact was performed using Graphpad (https://www.graphpad.com/quickcalcs/catMenu/). Also, the significance was calculated by comparing the proportions between two sample sizes using Epicalc 2000 Version 1.02 (J & Myatt M, Brixton Books, Brixton, UK). *P* ≤ 0.05 was considered to be statistically significant.

## Supplementary information

**Additional file 1: Table S1.** Distribution oc clonal complexes (CCs) among MRSA isolates 2016–2017.

## Data Availability

All relevant data are available in the manuscript and supplementary file.

## Abbreviations

MRSA: Methicillin-resistant *Staphylococcus aureus*
HA-MRSA: Healthcare-associated or healthcare-acquired MRSA
CA-MRSA: Community-associated or community-acquired MRSA
*spa*: Staphylococcal protein A
MLST: Multilocus sequence typing
PFGE: Pulsed-filed gel electrophoresis
CC: Clonal Complex
MIC: Minimum inhibitory concentration
